# Impact of Changes in Renal Function on Outcomes Following Mitral Transcatheter Edge-To-Edge Repair

**DOI:** 10.1016/j.shj.2025.100747

**Published:** 2025-10-29

**Authors:** Parth N. Patel, Olivia L. Hulme, Marc Allard-Ratick, Jay Khambhati, Amanda Stebbins, Andrzej S. Kosinski, Sreekanth Vemulapalli, Sammy Elmariah

**Affiliations:** a Division of Cardiology, Massachusetts General Hospital, Boston, Massachusetts, USA; b Division of Cardiology, Brigham & Women’s Hospital, Boston, Massachusetts, USA; c Duke University Medical Center, Duke Clinical Research Institute, Durham, North Carolina, USA; d Division of Cardiology, University of California-San Francisco, San Francisco, California, USA

**Keywords:** Chronic kidney disease, Mitral regurgitation, mTEER, Outcomes

## Abstract

**Background:**

Hemodynamic changes following mitral transcatheter edge-to-edge repair (mTEER) may impact estimated glomerular filtration rate (eGFR), but whether changes in eGFR following mTEER are associated with subsequent clinical outcomes is not known. The objective of the study was to investigate procedure-related changes in eGFR and clinical outcomes following mTEER.

**Methods:**

We studied patients in the Transcatheter Valve Therapy registry undergoing mTEER between 2013 and 2022 with available baseline and discharge eGFR. Multivariable linear regression identified baseline characteristics associated with changes in eGFR following mTEER. Cox proportional hazards models examined the adjusted association between improved (≥10% increase in eGFR), unchanged, and worsened renal function (≥10% decrease in eGFR) and survival at 1 year.

**Results:**

Among 48,472 patients undergoing mTEER, 15.7% experienced improved renal function and 13.7% had worsened renal function. Cardiogenic shock within 24 hours, age >80, Black race, diabetes, heart failure within 2 weeks, and female sex were strongly associated with worsened renal function after mTEER. Compared to the group with no change in renal function, improved renal function was associated with a decreased risk of 1-year mortality (adjusted hazard ratio: 0.77; 95% CI 0.69-0.86, *p* < 0.001), whereas worsened renal function was associated with an increased risk of death at 1 year (adjusted hazard ratio: 2.85; 95% CI 2.64-3.08, *p* < 0.001).

**Conclusions:**

Approximately 30% of patients experience marked changes in renal function following mTEER.

A greater than 10% increase or decrease in eGFR is independently associated with differences in 1-year survival. Careful attention to patients at greatest risk for worsened renal function after mTEER is warranted.

## Highlights

• In the Transcatheter Valve Therapy registry, 30% of patients experience a ±10% change in estimated glomerular filtration rate (eGFR) after mitral transcatheter edge-to-edge repair.
• Improvement in eGFR (+10%) is independently associated with better 1-year survival.
• Worsened eGFR (−10%) independently associates with a greater risk of 1-year mortality.
• Improvements in eGFR may be a key mediator of future clinical outcomes after mitral transcatheter edge-to-edge repair.

## Introduction

Chronic kidney disease (CKD) is a common comorbidity among patients undergoing cardiac surgical and transcatheter procedures.^1^, ^2^, ^3^ In patients with severe mitral regurgitation (MR) being evaluated for mitral transcatheter edge-to-edge repair (mTEER), the presence and severity of CKD at baseline is a well-established risk factor for increased morbidity and mortality in follow-up.^4^^,^^5^ Additionally, acute kidney injury (AKI) has been shown to complicate 15 to 20% of mTEER procedures, and several studies suggest that AKI is an independent predictor of adverse short- and long-term clinical outcomes.^6^^,^^7^

In patients with severe MR, elevated cardiac filling pressures may decrease cardiac output, increase venous congestion, and trigger neurohormonal cascades that contribute to worsening renal function.^8^^,^^9^ Treatment of MR may improve these parameters and in turn lessen consequences of the cardiorenal syndrome. Reduction in MR severity via mTEER has been shown to improve renal function in a subset of patients with advanced CKD at baseline.^10^^,^^11^ Given that improvements in hemodynamics may enhance renal function in some patients, further investigation into the relationship between mTEER, post-procedure renal function, and outcomes is warranted.

Using the Society of Thoracic Surgeons (STS) and American College of Cardiology Transcatheter Valve Therapy (TVT) Registry, we sought to describe the distribution, predictors, and outcomes of procedure-related changes in estimated glomerular filtration rate (eGFR) following mTEER in a large, real-world US-based cohort. Our goal was to describe the changes in eGFR manifested at hospital discharge and identify the baseline factors that predicted significant changes in renal function following mTEER. Additionally, we attempted to evaluate the association between post-procedure changes in eGFR and clinical outcomes at 1 year.

## Methods

### Study Cohort

The American College of Cardiology/STS TVT Registry is a national, mandated registry of all transcatheter valve procedures performed in the United States outside of a clinical trial setting, for which hospital participation is required for Medicare and Medicaid national coverage determination. TVT data collection forms include patient demographics, clinician and facility characteristics, history and risk factors, cardiac status, detailed health status, indications for the procedure, procedural data, adverse event rates, and outcomes at 30 days and 1 year.^12^ Given the obligatory requirement for data submission, the TVT Registry has captured nearly all mTEER procedures performed in the United States.^13^ The Duke Clinical Research Institute serves as one of the primary data analytic centers for research involving deidentified patient data. The registry has approval from a central institutional review board (Advarra) as well as a waiver of informed consent from the Duke University of School of Medicine.

The study population comprised all patients ≥18 years of age undergoing isolated mTEER procedures for moderate-severe or severe MR due to degenerative, functional, or mixed mitral valve disease pathologies in the TVT Registry from November 1, 2013, to March 31, 2022. Subjects with both baseline and predischarge eGFR reported within registry data were included in the analysis. Patients with baseline end-stage renal disease on hemodialysis, those with prior mTEER procedures, or patients who required reintervention within 30 days of the index procedure were excluded.

### Outcomes

The main objectives of this study were to describe changes in renal function following mTEER and to evaluate the association between postprocedure changes in eGFR and clinical outcomes. The primary endpoint was 1-year mortality. The secondary endpoint was the composite of mortality and heart failure (HF) hospitalization at 1 year. Additional postprocedure and in-hospital outcomes of interest included rates of successful device deployment, procedural success, cardiac arrest, cardiac perforation, unplanned cardiac surgery, Valve Academic Research Consortium major bleeding, new requirements for hemodialysis, and death. Valve Academic Research Consortium major bleeding was defined as a life-threatening or disabling bleed.^14^ Procedural success was defined as at least 1 device successfully deployed, relative reduction in MR of at least 1 grade, and postprocedure MR of none, trace, or mild.

### Definitions

Glomerular filtration rate values were estimated (eGFR) using the Chronic Kidney Disease Epidemiology Collaboration equation.^15^ Estimated GFR was calculated based on creatinine values reported at baseline (most recent measurement within 30 days before the procedure) and predischarge (last value before hospital discharge).

Subjects were classified into three groups based on change in eGFR from baseline (preprocedure) to predischarge. Patients with improvement in renal function were defined as those with ≥10% increase in eGFR following procedure. Patients with worsening of renal function were those with ≥10% decrease in eGFR following the procedure. Patients with <10% absolute change in eGFR in the periprocedural period were categorized as having no meaningful change in renal function. These thresholds were selected based on prior literature studying renal function after similar transcatheter procedures.^16^, ^17^, ^18^ Baseline renal function was categorized into five stages based on preprocedure eGFR values, as previously described.^19^

### Statistical Analysis

Summary statistics for the cohort are presented as means ± standard deviation (SD) or medians with interquartile range for continuous data and as percentages for categorical data. Continuous variables are compared using one-way analysis of variance. Categorical variables are compared using the chi-square test. Differences among baseline characteristics, postprocedure, and in-hospital outcomes were assessed across three groups: those with improvement in renal function, no change in renal function, or worsening of renal function.

To determine baseline characteristics associated with changes in renal function following mTEER, we fit a multivariable linear regression model to the analytic data set with change in eGFR as the continuous outcome.^20^ The generalized estimating equations were used to account for clustering of patients within sites. The prediction model was adjusted for a prespecified set of baseline factors which included age, sex, body mass index, race, Hispanic ethnicity, hemoglobin, baseline eGFR, diabetes, smoking status, peripheral artery disease, prior stroke, transient ischemic attack, chronic lung disease, home oxygen use, prior myocardial infarction, percutaneous coronary intervention, coronary artery bypass grafting, left main stenosis ≥50%, aortic stenosis, moderate-severe aortic insufficiency, prior aortic valve procedure, left ventricular ejection fraction, tricuspid insufficiency, prior tricuspid procedure, atrial fibrillation, prior pacemaker, endocarditis, etiology of mitral valve disease, HF within 2 weeks, and cardiogenic shock within 24 hours. Modeling assumptions were verified. For variables which were not linearly associated with changes in eGFR in the model (age, body mass index, left ventricular ejection fraction, hemoglobin), those measures were modeled as linear splines with inflection points placed where the linearity assumption was violated. These variables were modeled using more than 1 term. Baseline characteristics associated with changes in renal function are presented as point estimates for changes in eGFR with 95% CIs.

To assess the impact of changes in renal function following mTEER and 1-year clinical outcomes, we used Kaplan–Meier methods to estimate the cumulative incidence function and constructed Cox proportional hazards models adjusted for the same baseline characteristics as used in the multivariable linear regression analysis. Based on the observation that those who were critically ill at baseline were more likely to have worse procedural outcomes following mTEER, we performed two sensitivity analyses to better define the association between changes in renal function and outcomes in the survival analysis. First, we limited the cohort to patients undergoing elective procedures. Second, we performed an analysis in which outcomes were adjusted for the occurrence of severe procedural complications (cardiac arrest, cardiac perforation, major or life-threatening bleeding, unplanned cardiac or vascular surgical interventions) to account for the effect of procedural adversity on 1-year outcomes. For all models, only subjects in whom 425 days had elapsed between their procedure date and date of data harvest were eligible for the 1-year follow up analysis (75% of cohort). All modeling assumptions were tested and transformations performed when necessary. Results are presented as adjusted hazard ratios (aHR) with 95% CI.

## Results

### Study Cohort

**Figure 1:**
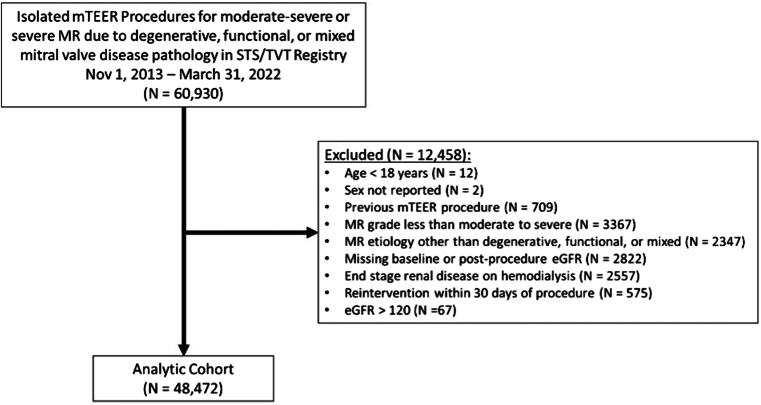
**Study cohort.** Inclusion and exclusion criteria used in creation of the final analytic cohort. Abbreviations: eGFR, estimated glomerular filtration rate; MR, mitral regurgitation; mTEER, mitral transcatheter edge-to-edge repair; STS, Society of Thoracic Surgeons; TVT, Transcatheter Valve Therapy.

Between November 1, 2013, and March 31, 2022, a total of 60,930 subjects underwent isolated mTEER in the STS/TVT Registry. After applying the inclusion and exclusion criteria, the final study cohort included 48,472 individuals (Figure 1). Approximately 5% of subjects (n = 2822) within the registry were excluded on the basis of missing baseline or pre-discharge eGFR values. Comparison of subjects with and without available eGFR data are shown in Supplementary Table S1.

The study population had a mean age of 77.8 ± 10.1 years, was 86.4% White, and 46.2% were female. The etiology of MR was degenerative in 69.0%, functional in 19.8%, and mixed in 11.2% of patients. The mean STS risk score was 6.4% ± 6.2%. The mean baseline eGFR was 57.7 ± 21.7 ml/min/1.73 m^2^. The proportion of patients with stage 1 (eGFR ≥90), stage 2 (eGFR 60-89), stage 3 (eGFR 30-59), stage 4 (eGFR 15-29), and stage 5 (eGFR <15) CKD at baseline was 7.8, 36.7, 45.2, 9.3, and 1.0%, respectively.

### Renal Function After mTEER

Mean predischarge eGFR was 57.9 ± 22.8 ml/min/1.73 m^2^. A total of 7615 subjects (15.7%) were found to have improved renal function (≥10% increase in eGFR), whereas 6628 (13.7%) had worsened renal function (≥10% decrease in eGFR) observed before hospital discharge. The baseline and predischarge eGFR of patients with improved, worsened, and no change in renal function following mTEER are shown in Supplementary Table S2. Among those who had improvement in renal function following mTEER, the mean increase in eGFR was 16.8 ± 7.2 ml/min/1.73 m^2^. In patients with worsened renal function, the mean decrease in eGFR was 20.1 ± 11.2 ml/min/1.73 m^2^. Only 21,259 patients (43.9%) in the analytic cohort had eGFR values available at 30 days.

### Predictors of Change in eGFR Following mTEER

Baseline characteristics of individuals with improved, no change, and worsened renal function following mTEER are displayed in Table 1. Patients with worsened renal function post procedure were more likely to be female and of non-White race, with 10.9% self-identifying as Black and 8.0% as Hispanic or Latino ethnicity. The group with worsened renal function had a higher rate of comorbidities such as diabetes, chronic lung disease, peripheral arterial disease, and HF. On clinical presentation, patients in the worsened renal function group more frequently had New York Heart Association functional class IV symptoms, HF symptoms within the 2 weeks prior, cardiogenic shock in the preceding 24 hours, and current inotrope dependence. Accordingly, a greater proportion of procedures in those who had worsened renal function post procedure were performed urgently or emergently. Individuals with worsened renal function were more likely to have isolated functional MR and less likely to have purely degenerative mitral pathology.

**Table 1:** Baseline characteristics of study cohort

Characteristic	All patients (N = 48,472)	Improved renal function (N = 7615)	Unchanged renal function (N = 34,229)	Worsened renal function (N = 6628)	*p* Value
Demographics					
Age (yrs)	77.8 ± 10.1	77.6 ± 9.8	78.2 ± 9.8	76.0 ± 11.3	<0.0001
Female sex	22,408 (46.2%)	3580 (47.0%)	15,414 (45.0%)	3414 (51.5%)	<0.0001
Body mass index (kg/sqm)	26.8 ± 6.4	26.4 ± 6.0	26.8 ± 6.3	27.3 ± 7.1	<0.0001
Body surface area (sqm)	1.9 ± 0.3	1.8 ± 0.3	1.9 ± 0.3	1.8 ± 0.3	0.0009
Race/ethnicity					
White	41,875 (86.4%)	6758 (88.7%)	29,589 (86.4%)	5528 (83.4%)	<0.0001
Black	4254 (8.8%)	550 (7.2%)	2982 (8.7%)	722 (10.9%)	<0.0001
Asian	1125 (2.3%)	140 (1.8%)	807 (2.4%)	178 (2.7%)	0.0026
American Indian/Alaskan Native	175 (0.4%)	21 (0.3%)	123 (0.4%)	31 (0.5%)	0.1622
Native Hawaiian/Pacific Islander	116 (0.2%)	8 (0.1%)	90 (0.3%)	18 (0.3%)	0.0327
Hispanic/Latino ethnicity	2883 (6.0%)	424 (5.6%)	1936 (5.7%)	523 (8.0%)	<0.0001
Baseline KCCQ score					<0.0001
Mean ± SD	42.9 ± 24.9	46.0 ± 24.1	43.3 ± 24.9	37.2 ± 24.8	
Median (25th, 75th)	40.6 (23.4, 60.4)	43.8 (27.1, 63.5)	41.2 (24.0, 61.5)	33.3 (17.2, 54.2)	
STS 2007 risk model score (%)					<0.0001
Mean ± SD	6.4 ± 6.2	5.3 ± 4.7	6.5 ± 6.3	6.5 ± 7.0	
Median (25th, 75th)	4.7 (2.7, 7.8)	4.1 (2.5, 6.6)	4.8 (2.8, 8.1)	4.6 (2.5, 8.0)	
Baseline measures of renal function					
eGFR (CKD-EPI equation)					<0.0001
Mean ± S.D.	57.7 ± 21.7	56.3 ± 15.7	56.1 ± 22.7	67.7 ± 19.6	
Median (25th, 75th)	56.6 (41.2, 74.0)	56.6 (45.5, 68.3)	54.0 (38.4, 73.3)	68.3 (53.2, 84.2)	
CKD stage					<0.0001
Stage 1 [eGFR ≥ 90]	3801 (7.8%)	49 (0.6%)	2895 (8.5%)	857 (12.9%)	
Stage 2 [eGFR 60-89]	17,774 (36.7%)	3140 (41.2%)	11,259 (32.9%)	3375 (50.9%)	
Stage 3 [eGFR 30-59]	21,887 (45.2%)	4094 (53.8%)	15,566 (45.5%)	2227 (33.6%)	
Stage 4 [eGFR 15-29]	4523 (9.3%)	265 (3.5%)	4089 (11.9%)	169 (2.5%)	
Stage 5 [eGFR <15]	487 (1.0%)	67 (0.9%)	420 (1.2%)	0 (0.0%)	
History and risk factors					
Hypertension	41,745 (86.2%)	6506 (85.5%)	29,572 (86.4%)	5667 (85.5%)	0.0231
Diabetes	13,508 (27.9%)	1752 (23.0%)	9714 (28.4%)	2042 (30.8%)	<0.0001
Prior MI	12,683 (26.2%)	1859 (24.4%)	9011 (26.4%)	1813 (27.4%)	0.0002
Atrial fibrillation/flutter	30,354 (62.7%)	4789 (63.0%)	21,381 (62.5%)	4184 (63.2%)	0.5000
Prior stroke	5289 (10.9%)	788 (10.4%)	3736 (10.9%)	765 (11.5%)	0.0741
PAD	7799 (16.1%)	1113 (14.6%)	5583 (16.3%)	1103 (16.7%)	0.0005
Chronic lung disease	16,898 (35.1%)	2588 (34.2%)	11,745 (34.5%)	2565 (39.0%)	<0.0001
Home oxygen use	5220 (10.8%)	715 (9.4%)	3630 (10.6%)	875 (13.2%)	<0.0001
Immunocompromise present	3494 (7.5%)	507 (6.9%)	2482 (7.5%)	505 (7.9%)	0.0722
Current smoker	3439 (7.1%)	524 (6.9%)	2264 (6.6%)	651 (9.9%)	<0.0001
Procedure history					
Prior PCI	15,073 (31.1%)	2250 (29.6%)	10,765 (31.5%)	2058 (31.1%)	0.0050
Coronary artery bypass graft	11,194 (23.1%)	1733 (22.8%)	8091 (23.7%)	1370 (20.7%)	<0.0001
Pacemaker	9052 (18.7%)	1407 (18.5%)	6456 (18.9%)	1189 (18.0%)	0.1823
ICD	8531 (17.6%)	1289 (16.9%)	6096 (17.8%)	1146 (17.3%)	0.1434
Prior mitral valve procedure	607 (1.3%)	81 (1.1%)	441 (1.3%)	85 (1.3%)	0.2682
Prior aortic valve procedure	4459 (9.2%)	629 (8.3%)	3209 (9.4%)	621 (9.4%)	0.0084
Presentation features					
Heart failure (w/in 2 wk)	38,194 (81.4%)	5838 (79.3%)	26,874 (81.1%)	5482 (85.4%)	<0.0001
Cardiogenic shock (w/in 24 h)	1041 (2.1%)	63 (0.8%)	595 (1.7%)	383 (5.8%)	<0.0001
NYHA class (w/in 2 wk)					<0.0001
I/II	8751 (18.2%)	1560 (20.7%)	6317 (18.6%)	874 (13.3%)	
III/IV	39,281 (81.8%)	5991 (79.4%)	27,606 (81.4%)	5684 (86.7%)	
Procedure acuity					<0.0001
Elective	40,392 (83.3%)	6922 (90.9%)	28,794 (84.1%)	4676 (70.5%)	
Urgent	4525 (9.3%)	334 (4.4%)	3155 (9.2%)	1036 (15.6%)	
Preprocedural shock/inotropes or mech assist device	3045 (6.3%)	322 (4.2%)	1979 (5.8%)	744 (11.2%)	
Emergent/salvage/prior cardiac arrest w/in 24h	510 (1.1%)	37 (0.5%)	301 (0.9%)	172 (2.6%)	
Baseline echocardiogram					
LVEF (%)					<0.0001
Mean ± S.D.	47.9 ± 15.4	48.5 ± 15.1	48.0 ± 15.3	46.6 ± 16.3	
Median (25th, 75th)	53.0 (35.0, 60.0)	54.0 (37.0, 60.0)	53.0 (35.0, 60.0)	50.0 (33.0, 60.0)	
Mitral regurgitation severity					0.3811
Moderate/severe	8986 (18.5%)	1437 (18.9%)	6357 (18.6%)	1192 (18.0%)	
Severe	39,486 (81.5%)	6178 (81.1%)	27,872 (81.4%)	5436 (82.0%)	
Etiology of mitral regurgitation					
Functional mitral valve etiology	9621 (19.8%)	1392 (18.3%)	6781 (19.8%)	1448 (21.8%)	<0.0001
Degenerative mitral valve etiology	33,438 (69.0%)	5462 (71.7%)	23,586 (68.9%)	4390 (66.2%)	<0.0001
Mixed degenerative/functional	5413 (11.2%)	761 (10.0%)	3862 (11.3%)	790 (11.9%)	0.0006
Tricuspid regurgitation severity					<0.0001
None/trace/mild	24,607 (51.1%)	3962 (52.4%)	17,446 (51.4%)	3199 (48.6%)	
Moderate	16,109 (33.5%)	2567 (33.9%)	11,372 (33.5%)	2170 (33.0%)	
Severe	7421 (15.4%)	1037 (13.7%)	5170 (15.2%)	1214 (18.4%)	

Abbreviations: CKD, Chronic kidney disease; CKD-EPI, Chronic Kidney Disease Epidemiology Collaboration; eGFR, estimated glomerular filtration rate; ICD, International Classification of Diseases; KCCQ, Kansas City Cardiomyopathy Questionnaire; LVEF, left ventricular ejection fraction; MI, myocardial infarction; NYHA, New York Heart Association; PAD, peripheral artery disease; PCI, percutaneous coronary intervention; STS, Society of Thoracic Surgeons.

In the multivariable analysis, several baseline factors independently predicted change in eGFR after mTEER (Table 2). Cardiogenic shock within 24 hours of the procedure was the strongest predictor of changes in renal function following the procedure, associated with a −7.52 ml/min/1.73 m^2^ (95% CI: −8.47 to −6.58, *p* < 0.001) decrease in eGFR before discharge. Other factors significantly associated with worsened renal function included age >80, Black race, female sex, diabetes mellitus, and HF within 2 weeks of the procedure (*p* < 0.001 for all). In contrast, higher hemoglobin levels were associated with improved renal function after mTEER, especially among those with baseline hemoglobin levels ≤12.5 g/dl (5.62 ml/min/1.73 m^2^ increase in eGFR per 5 g/dl unit change, 95% CI: 4.41 to 6.84, *p* < 0.001).

**Table 2:** Characteristics independently associated with changes in eGFR after mTEER

	Estimate (95% CI) mL/min/1.73 m2	*p* value
Cardiogenic shock (within 24 h)	−7.52 (−8.47, −6.58)	<0.001
Age >80 [10 y]	−1.83 (−2.14, −1.51)	<0.001
Baseline GFR [10 unit change]	−1.38 (−1.45, −1.31)	<0.001
Race [Black vs non-Black]	−1.23 (−1.61, −0.85)	<0.001
Diabetes	−1.22 (−1.50, −0.94)	<0.001
Heart failure within 2 wk of procedure	−1.05 (−1.42, −0.68)	<0.001
Female sex	−0.81 (−1.04, −0.58)	<0.001
BMI >20 [5 unit change]	−0.68 (−0.78, −0.57)	<0.001
LVEF >60 [10 unit change]	−0.68 (−1.04, −0.31)	<0.001
Prior PAD	−0.65 (−0.93, −0.37)	<0.001
Hispanic ethnicity	−0.64 (−1.13, −0.15)	0.011
Moderate/severe tricuspid valve insufficiency	−0.62 (−0.85, −0.40)	<0.001
Home oxygen use	−0.56 (−0.92, −0.21)	0.002
Chronic lung disease	−0.44 (−0.66, −0.22)	<0.001
Prior aortic valve procedure	−0.38 (−0.76, −0.00)	0.048
Prior stroke	−0.37 (−0.70, −0.04)	0.027
Prior MI	−0.26 (−0.51, −0.02)	0.034
Age ≤ 80 [10 y]	−0.21 (−0.41, −0.01)	0.037
Prior CABG	0.47 (0.21, 0.72)	<0.001
LVEF ≤ 60 [10 unit change]	0.48 (0.38, 0.58)	<0.001
Hgb >12.5 [5 unit change]	3.00 (2.19, 3.82)	<0.001
Hgb ≤ 12.5 [5 unit change]	5.62 (4.41, 6.84)	<0.001

Abbreviations: BMI, body mass index; CABG, coronary artery bypass grafting; eGFR, estimated glomerular filtration rate; GFR, glomerular filtration rate; Hgb, hemoglobin; LVEF, left ventricular ejection fraction; MI, myocardial infarction; PAD, peripheral artery disease; mTEER, mitral transcatheter edge-to-edge repair.

### Procedural and In-Hospital Outcomes

Procedural success was less likely to be observed in individuals with postprocedural worsened renal function (58.4%) when compared to those with unchanged (64.4%) or improved (67.4%) renal function (Table 3). In patients with subsequent worsening of renal function, the procedure was more likely to be complicated by major or life-threatening bleeding, cardiac arrest, cardiac perforation, or unplanned cardiac or vascular surgical interventions. These same individuals were more likely to require initiation of dialysis during the index hospitalization. Unadjusted in-hospital mortality rates differed across the three groups of patients and were 7.3% in the worsened renal function group, 0.9% in the no change group, and 0.3% in the improved renal function group. Comprehensive procedural outcomes for the cohort are presented in Supplementary Table S3.

**Table 3:** Procedural and in-hospital outcomes by changes in renal function

Characteristic	All patients (N = 48,472)	Improved Renal function (N = 7615)	Unchanged renal function (N = 34,229)	Worsened Renal function (N = 6628)	*p* value
Number of successfully deployed devices	1.4 ± 0.6	1.4 ± 0.6	1.4 ± 0.6	1.5 ± 0.7	<0.0001
Procedural success∗	31,035 (64.0%)	5132 (67.4%)	22,035 (64.4%)	3868 (58.4%)	<0.0001
Mitral valve gradient (mean-mm Hg)	3.6 ± 2.0	3.4 ± 1.9	3.5 ± 2.0	3.8 ± 2.2	<0.0001
Postprocedure mitral regurgitation					<0.0001
None	615 (1.3%)	110 (1.5%)	431 (1.3%)	74 (1.2%)	
Trace/trivial	8007 (17.3%)	1351 (18.5%)	5752 (17.6%)	904 (14.3%)	
Mild (1+)	25,880 (55.8%)	4214 (57.8%)	18,407 (56.2%)	3259 (51.6%)	
Moderate (2+)	9798 (21.1%)	1381 (18.9%)	6860 (20.9%)	1557 (24.6%)	
Moderate-severe (3+)	740 (1.6%)	95 (1.3%)	502 (1.5%)	143 (2.3%)	
Severe (3/4+)	434 (0.9%)	44 (0.6%)	276 (0.8%)	114 (1.8%)	
Severe (4+)	911 (2.0%)	101 (1.4%)	539 (1.6%)	271 (4.3%)	
Cardiac arrest	459 (0.9%)	23 (0.3%)	217 (0.6%)	219 (3.3%)	<0.0001
Cardiac perforation	322 (0.7%)	35 (0.5%)	175 (0.5%)	112 (1.7%)	<0.0001
Cardiac surgery, unplanned	664 (1.4%)	47 (0.6%)	345 (1.0%)	272 (4.1%)	<0.0001
Access site bleed	334 (0.7%)	37 (0.5%)	229 (0.7%)	68 (1.0%)	0.0004
In-hospital VARC degree of bleeding					<0.0001
No VARC bleeding	47,056 (97.1%)	7479 (98.2%)	33,416 (97.6%)	6161 (93.0%)	
Major bleed (not life threatening)	920 (1.9%)	106 (1.4%)	571 (1.7%)	243 (3.7%)	
Life-threatening or disabling bleed	488 (1.0%)	28 (0.4%)	238 (0.7%)	222 (3.4%)	
Postprocedure AKI					<0.0001
No AKI	43,440 (89.6%)	7613 (100.0%)	32,772 (95.7%)	3055 (46.1%)	
Stage 1	4504 (9.3%)	0 (0.0%)	1296 (3.8%)	3208 (48.4%)	
Stage 2	86 (0.2%)	0 (0.0%)	0 (0.0%)	86 (1.3%)	
Stage 3	442 (0.9%)	2 (0.0%)	161 (0.5%)	279 (4.2%)	
Dialysis -new requirement	315 (0.6%)	2 (0.0%)	108 (0.3%)	205 (3.1%)	<0.0001
In-hospital death	799 (1.6%)	21 (0.3%)	293 (0.9%)	485 (7.3%)	<0.0001

Abbreviations: AKI, acute kidney injury; MR, mitral regurgitation; VARC, Valve Academic Research Consortium.

∗ Procedural success is defined as at least 1 device was successfully deployed, procedure was not aborted, relative reduction in MR of at least 1 grade and post procedure mitral regurgitation of none, trace/trivial or mild.

### 1-Year Clinical Outcomes

**Figure 2:**
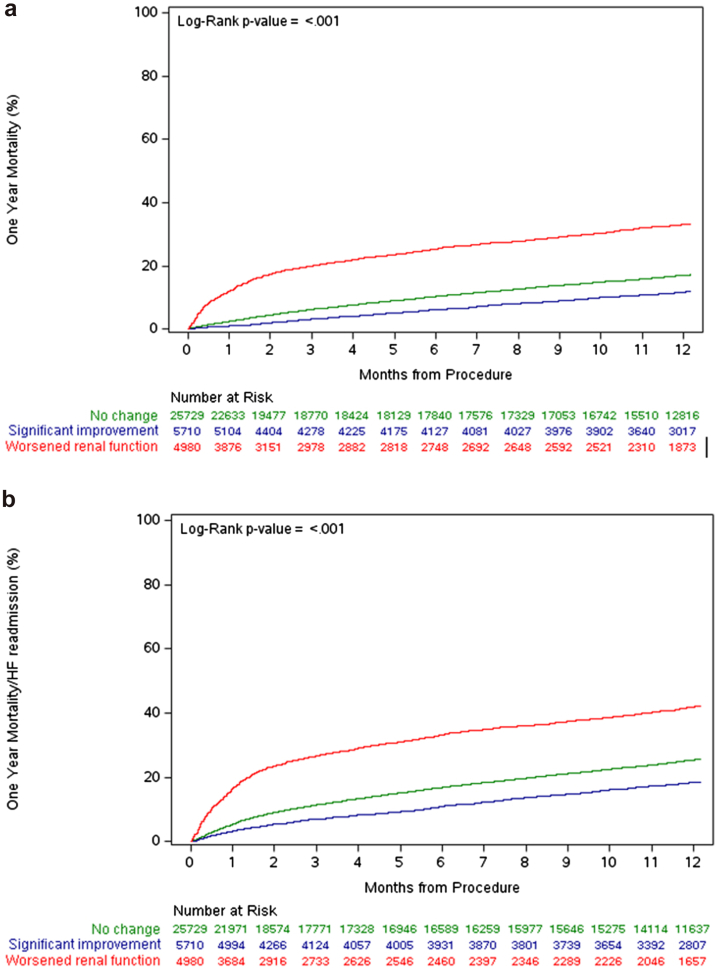
**Kaplan–Meier estimates of 1-year clinical outcomes by changes in renal function.** Cumulative incidence of (a) all-cause mortality and (b) all-cause mortality and heart failure hospitalization are shown. Number at risk reflects the cohort of subjects with 1 year follow-up data available. Abbreviation: HF, heart failure.

The cumulative incidence of death at 1 year was 11.9% in the improved renal function group, 17.2% in the unchanged renal function group, and 33.3% in the worsened renal function group (Figure 2a). After adjusting for clinical risk factors, improved renal function following mTEER remained independently associated with a lower risk of 1-year mortality compared to unchanged renal function (aHR 0.77, 95% CI: 0.69 to 0.86, *p* < 0.001). By contrast, when compared to the patients who experienced no change in renal function, those with worsened renal function had a nearly 3-fold increase in the risk of death at 1 year in the fully adjusted model (aHR 2.85, 95% CI: 2.64 to 3.08, *p* < 0.001, Table 4).

**Table 4:** Associations between changes in renal function following mTEER and 1-y clinical outcomes

	Event rate	Adjusted HR (95% CI)	*p* value
All-cause mortality (1 y)			
Improved renal function	11.9%	0.77 (0.69, 0.86)	<0.001
Unchanged renal function	17.2%	Reference	--
Worsened renal function	33.3%	2.85 (2.64, 3.08)	<0.001
All-cause mortality and HF hospitalization (1 y)			
Improved renal function	18.6%	0.79 (0.72, 0.86)	<0.001
Unchanged renal function	25.7%	Reference	--
Worsened renal function	42.3%	2.27 (2.12, 2.43)	<0.001

Abbreviations: HF, heart failure; HR, hazard ratio; mTEER, mitral transcatheter edge-to-edge repair.

The cumulative incidence of the secondary composite endpoint of death or HF hospitalization at 1 year was 18.6% in the improved renal function group, 25.7% in the unchanged renal function group, and 42.3% in the worsened renal function group (Figure 2b). Improvement in renal function was similarly associated with a decreased risk of the composite endpoint (aHR 0.79, 95% CI: 0.72 to 0.86, *p* < 0.001), whereas increased hazard was observed in those who had worsening of renal function following mTEER (aHR 2.27, 95% CI: 2.12 to 2.43, *p* < 0.001, Table 4).

In order to assess the robustness of our findings, we performed two sensitivity analyses to further define the association between changes in renal function and 1-year clinical outcomes. First, the association between changes in renal function and 1-year outcomes was investigated in the subset of patients undergoing elective procedures. Second, the model was adjusted for the occurrence of severe procedural complications (cardiac arrest, cardiac perforation, major or life-threatening bleeding, unplanned cardiac or vascular surgical interventions) to determine whether the association between post-procedure renal function and outcomes was independent of procedural adversity. In both sensitivity analyses, the findings remained consistent (Supplementary Table S4).

## Discussion

In this TVT registry analysis of nearly all mTEER procedures performed in the United States, we observed significant variability in renal response to mTEER, with approximately 30% of patients experiencing ≥10% changes in eGFR by the time of hospital discharge. A ≥10% worsening of eGFR in the postprocedure period occurred in 14% of patients and was independently associated with a nearly 3-fold increased risk of death at 1 year. In contrast, a ≥10% improvement in eGFR following mTEER was observed in 16% and remained independently associated with a decreased risk for mortality at 1 year after adjusting for baseline characteristics. Patients presenting with cardiogenic shock in the 24 hours before the procedure were at the greatest risk for a decrease in eGFR following mTEER. Improvements in eGFR were more likely to be seen in patients with higher hemoglobin levels at baseline.

Whether the treatment of MR can positively affect cardiorenal physiology to improve clinical outcomes remains poorly established. In the present analysis, we demonstrate that about 15% of patients experience a notable improvement in renal function following mTEER, with a median increase in eGFR of 14.7 ml/min/1.73 m^2^ postprocedure. Furthermore, this cohort of patients had improved survival at 1 year, even when compared to the group of patients in whom renal function remained unchanged. Taken together, these findings support the notion that treatment of MR has the potential to improve the cardiorenal syndrome in some patients, and that changes in cardiorenal physiology may be a key mediator of future clinical outcomes following mTEER. In the present analysis, the group which experienced an improvement in renal function had more advanced stages of CKD at baseline, a finding that is consistent with previous data reported across several smaller studies.^9^^,^^10^^,^^21^ The validation that those with greatest baseline renal dysfunction may potentially derive the largest benefit from mitral valve intervention in this national registry highlights the critical need to better understand the cardiorenal syndrome and identify the patients in whom renal recovery remains possible.

Conversely, our findings demonstrate that worsened renal function persists at hospital discharge in 14% of patients following mTEER. This incidence is similar to the 15% rate of AKI after mTEER in previously published studies.^7^ Although mTEER procedures are generally free of iodinated contrast use, our study underscores the underappreciated but inherent risk for deteriorating renal function following mTEER intervention. In patients who experienced worsened renal function, we observed that the procedure was more likely to be complicated by major or life-threatening bleeding, cardiac arrest, cardiac perforation, or unplanned cardiac or vascular surgical interventions. In addition, procedural success was less likely to be observed in this cohort. Further research is needed to better understand the patients in whom mTEER is most likely to be successful, as procedure-related complications and subsequent worsening of renal function appear to confer a greater risk of 1-year mortality.

Those who experienced worsened renal function postprocedure were more likely to have a higher burden of baseline comorbidities. In addition, these patients appeared to have a profile on presentation representing advanced HF and/or greater hemodynamic instability in the period before intervention. In addition to preprocedure cardiogenic shock within 24 hours being predictive of an adverse change in renal function, we identified female sex, Black race, diabetes, and moderate to severe tricuspid insufficiency as additional factors associated with a decrease in eGFR following mTEER. Consistent with prior work,^9^^,^^22^, ^23^, ^24^, ^25^, ^26^ we demonstrate a possible protective effect of higher hemoglobin levels against worsening renal function. While the absence of anemia may reflect patients with less advanced CKD and more resilient renal function, lower hemoglobin levels may also predispose individuals to a higher burden of hypoxia and oxidative stress in the peri-procedural period. Furthermore, red blood cell transfusion has been strongly linked to a higher risk of AKI in other similar settings,^27^^,^^28^ and is likely to be more frequent in those with more severe anemia before mTEER. Although the majority of baseline factors identified in this study are not immediately modifiable in the short term, they nonetheless serve as risk markers for which preprocedure optimization of cardiogenic shock, volume overload, or anemia may preserve or enhance renal function following mTEER.

Finally, although the median change in eGFR was around 15 ml/min/1.73 m^2^ in the groups with a ≥10% change in eGFR by hospital discharge, these changes were attenuated in the subset of our cohort who had 30-day creatinine values available. In fact, approximately 25% of each group were observed to have complete return to preprocedural baseline values at 30 days, suggesting that eGFR at 30 days may better reflect a patient’s new steady-state that incorporates the effect of both periprocedural stressors as well as improved hemodynamics following mTEER. Further research into optimal management pathways which lead to durable treatment of MR, preserved cardiac hemodynamics, enhanced renal function, and improved clinical outcomes is needed.

## Limitations

This was a retrospective, observational study using the TVT registry in which data are site reported. Despite rigorous efforts at quality assurance, data completeness, and site-level auditing, the observed association of renal function on outcomes may be confounded by unmeasured variables. However, the results of this present analysis remain consistent with prior studies which link advanced renal dysfunction to adverse postprocedural outcomes. In addition, 30-day creatinine was not widely available in this registry, which may ultimately represent a more accurate estimate of steady-state renal function following mTEER. Our dataset does not quantify the degree of albuminuria or proteinuria at baseline, which may be important modifiers of renal risk and cardiovascular outcomes. The TVT registry has high rates of missingness with respect to echocardiographic follow-up, so it is not possible to assess whether the durability in mitral repair was different in those with improved versus worsened renal function. Lastly, decreases in eGFR at time of hospital discharge may result in the deprescription of guideline-directed medical therapy for HF. Our data source was not able to determine the ways in which this practice may have contributed to long-term risks.

## Conclusions

In conclusion, despite the lack of iodinated contrast use during mTEER, meaningful changes in renal function, both improvement and worsening, are often observed. Improvement in renal function after mTEER results in improved 1-year survival; whereas worsened renal function associated with increased risk of mortality. Several patient-level features may identify those at an increased risk for deterioration of renal function following the procedure. Best management practices for these cohorts warrant further investigation and represent an opportunity for improvement.

## Ethics Statement

The authors confirm that all research reported has adhered to the relevant ethical guidelines. The TVT registry has approval from a central institutional review board (Advarra) as well as a waiver of informed consent from the Duke University School of Medicine.

## Funding

Dr Elmariah receives research funds to the institution and consulting fees from 10.13039/100006520Edwards Lifesciences, 10.13039/100011949Abbott Vascular, and 10.13039/100004374Medtronic.

## Disclosure Statement

Sammy Elmariah reports a relationship with Edwards Lifesciences Corporation that includes consulting or advisory and funding grants; a relationship with Medtronic that includes funding grants; a relationship with Abbott Vascular Inc that includes funding grants.

The other authors had no conflicts to declare.
