# Supplementary material for: Impact of Changes in Renal Function on Outcomes Following Mitral Transcatheter Edge-To-Edge Repair
Source: Struct Heart. 2025 Oct 29;10(3):100747. doi: 10.1016/j.shj.2025.100747 (PMC12890816; doi:10.1016/j.shj.2025.100747)
Supplement: Supplementary Tables S1-S4 [file mmc1.docx]

**Supplemental Tables**

**Supplemental Table 1: Baseline Characteristics of Patients Included and Excluded on Basis of Available eGFR Values**

| **Characteristic** | **All Patients (N=54513)** | **eGFR not reported (N=2822)** | **eGFR reported (N=51691)** | **P value** |
| --- | --- | --- | --- | --- |
| **Demographics** |  |  |  |  |
| Age (yrs) | 77.4 +/- 10.3 | 77.2 +/- 10.3 | 77.4 +/- 10.3 | 0.3665 |
| Female Sex | 25061(46.0%) | 1338(47.4%) | 23723(45.9%) | 0.1109 |
| Body Mass Index (kg/sqm) | 26.8 +/- 6.4 | 27.1 +/- 6.4 | 26.8 +/- 6.4 | 0.0264 |
| Race/Ethnicity |  |  |  |  |
| White | 46668(85.6%) | 2454(87.0%) | 44214(85.5%) | 0.0358 |
| Black | 5066(9.3%) | 199(7.1%) | 4867(9.4%) | <.0001 |
| American Indian/Alaskan Native | 207(0.4%) | 10(0.4%) | 197(0.4%) | 0.8220 |
| Asian | 1362(2.5%) | 85(3.0%) | 1277(2.5%) | 0.0727 |
| Native Hawaiian/Pacific Islander | 142(0.3%) | 8(0.3%) | 134(0.3%) | 0.8056 |
| Hispanic or Latino Ethnicity | 3362(6.2%) | 142(5.1%) | 3220(6.3%) | 0.0104 |
| Baseline KCCQ Score |  |  |  | <.0001 |
| Mean +/- S.D. | 42.9 +/- 24.9 | 46.8 +/- 24.6 | 42.7 +/- 24.9 |  |
| Median (25th, 75th) | 40.6 (23.4, 60.9) | 44.8 (28.0, 64.8) | 40.6 (22.9, 60.4) |  |
| STS 2007 Risk Model Score (%) |  |  |  | <.0001 |
| Mean +/- S.D. | 6.9 +/- 7.4 | 5.1 +/- 5.0 | 7.0 +/- 7.4 |  |
| Median (25th, 75th) | 4.8 (2.8, 8.2) | 3.9 (2.2, 6.4) | 4.8 (2.8, 8.3) |  |
| **Baseline Measures of Renal Function** |  |  |  |  |
| eGFR (CKD EPI) |  |  |  | <.0001 |
| Mean +/- S.D. | 56.3 +/- 23.0 | 60.7 +/- 21.5 | 56.0 +/- 23.0 |  |
| Median (25th, 75th) | 55.6 (39.6, 73.4) | 59.3 (44.1, 77.8) | 55.4 (39.3, 73.2) |  |
| **History and Risk Factors** |  |  |  |  |
| Hypertension | 46966(86.2%) | 2404(85.2%) | 44562(86.2%) | 0.1357 |
| Diabetes | 15507(28.5%) | 723(25.6%) | 14784(28.6%) | 0.0006 |
| Prior MI | 14255(26.2%) | 598(21.2%) | 13657(26.4%) | <.0001 |
| Atrial Fibrillation/Flutter | 33762(62.0%) | 1677(59.5%) | 32085(62.1%) | 0.0047 |
| Prior Stroke | 5925(10.9%) | 297(10.5%) | 5628(10.9%) | 0.5426 |
| PAD | 8875(16.3%) | 401(14.2%) | 8474(16.4%) | 0.0023 |
| Chronic Lung Disease | 19015(35.1%) | 923(32.9%) | 18092(35.2%) | 0.0106 |
| Home Oxygen Use | 5894(10.8%) | 273(9.7%) | 5621(10.9%) | 0.0484 |
| Immunocompromise Present | 4003(7.6%) | 156(5.8%) | 3847(7.7%) | 0.0002 |
| Current Smoker | 3896(7.2%) | 186(6.6%) | 3710(7.2%) | 0.2404 |
| **Procedure History** |  |  |  |  |
| Prior PCI | 16900(31.0%) | 771(27.4%) | 16129(31.2%) | <.0001 |
| Coronary artery bypass graft | 12603(23.2%) | 598(21.2%) | 12005(23.3%) | 0.0136 |
| Pacemaker | 10053(18.5%) | 505(17.9%) | 9548(18.5%) | 0.4536 |
| ICD | 9474(17.4%) | 434(15.4%) | 9040(17.5%) | 0.0041 |
| Prior Mitral Valve Procedure | 1275(2.3%) | 45(1.6%) | 1230(2.4%) | 0.0071 |
| Prior Aortic Valve Procedure | 5044(9.3%) | 257(9.1%) | 4787(9.3%) | 0.7867 |
| **Presentation Features** |  |  |  |  |
| Heart Failure (w/in 2 wks) | 42874(81.3%) | 2030(75.2%) | 40844(81.6%) | <.0001 |
| Cardiogenic Shock (w/in 24 hrs) | 1241(2.3%) | 21(0.7%) | 1220(2.4%) | <.0001 |
| NYHA Class (w/in 2 wks) |  |  |  |  |
| I/II | 9840 (18.2%) | 633 (22.8%) | 9207 (18.0%) | <.0001 |
| III/IV | 44158 (81.8%) | 2141 (77.2%) | 42017 (82%) |  |
| Procedure Acuity |  |  |  | <.0001 |
| Elective | 45405(83.3%) | 2623(92.9%) | 42782(82.8%) |  |
| Urgent | 5041(9.2%) | 84(3.0%) | 4957(9.6%) |  |
| Pre-proc shock/inotropes or mech assist device | 3460(6.3%) | 98(3.5%) | 3362(6.5%) |  |
| Emergent/salvage/prior cardiac arrest w/in 24h | 607(1.1%) | 17(0.6%) | 590(1.1%) |  |

**Supplemental Table 2: Baseline and Post-Procedure eGFR Values by Changes in Renal Function**

|  | **All Patients** | **Improved**  **Renal Function**  **At Discharge** | **No**  **Change at Discharge** | **Worsened**  **Renal Function at Discharge** | **P value** |
| --- | --- | --- | --- | --- | --- |
| **Analytic Cohort** |  |  |  |  |  |
| N | 48472 | 7615 | 33229 | 6628 |  |
| Baseline eGFR |  |  |  |  | <.0001 |
| Mean +/- S.D. | 57.7 +/- 21.7 | 56.3 +/- 15.7 | 56.1 +/- 22.7 | 67.7 +/- 19.6 |  |
| Median (25th, 75th) | 56.6 (41.2, 74.0) | 56.6 (45.5, 68.3) | 54.0 (38.4, 73.3) | 68.3 (53.2, 84.2) |  |
| Post Procedure eGFR |  |  |  |  | <.0001 |
| Mean +/- S.D. | 57.9 +/- 22.8 | 73.1 +/- 15.9 | 56.5 +/- 23.0 | 47.7 +/- 20.1 |  |
| Median (25th, 75th) | 57.3 (40.3, 75.6) | 74.4 (61.5, 85.8) | 54.6 (38.6, 73.9) | 48.0 (32.6, 63.2) |  |
| Change in eGFR |  |  |  |  | <.0001 |
| Mean +/- S.D. | 0.2 +/- 11.9 | 16.8 +/- 7.2 | 0.4 +/- 4.8 | -20.1 +/- 11.2 |  |
| Median (25th, 75th) | 0.0 (-4.7, 6.4) | 14.7 (12.1, 19.2) | 0.0 (-2.8, 3.9) | -16.3 (-23.5, -12.6) |  |
| **30 Day Follow Up Cohort** |  |  |  |  |  |
| N | 21259 | 3362 | 15253 | 2644 |  |
| 30 Day eGFR |  |  |  |  | <.0001 |
| Mean +/- S.D. | 56.6 +/- 21.4 | 61.7 +/- 17.2 | 54.9 +/- 22.1 | 59.7 +/- 21.1 |  |
| Median (25th, 75th) | 55.2 (40.3, 72.3) | 61.1 (49.5, 73.7) | 52.7 (37.8, 71.1) | 59.3 (44.2, 74.8) |  |
| Change in eGFR |  |  |  |  |  |
| Mean +/- S.D. | -0.3 +/- 12.9 | 5.7 +/- 13.4 | -0.2 +/- 11.5 | -8.4 +/- 15.4 | <.0001 |
| Median (25th, 75th) | 0.0 (-6.7, 6.2) | 5.1 (-1.9, 12.8) | 0.0 (-5.9, 5.3) | -7.8 (-17.0, 0.0) |  |

**Supplemental Table 3: Complete Procedural and In-Hospital Outcomes by Changes in Renal Function**

| **Characteristic** | **All Patients (N=48472)** | **Improved**  **Renal Function (N=7615)** | **No**  **change (N=34229)** | **Worsened Renal Function (N=6628)** | **P value** |
| --- | --- | --- | --- | --- | --- |
| Number of successfully deployed devices | 1.4 +/- 0.6 | 1.4 +/- 0.6 | 1.4 +/- 0.6 | 1.5 +/- 0.7 | <.0001 |
| Procedural Success* | 31035(64.0%) | 5132(67.4%) | 22035(64.4%) | 3868(58.4%) | <.0001 |
| MV Gradient (mean-mm HG) | 3.6 +/- 2.0 | 3.4 +/- 1.9 | 3.5 +/- 2.0 | 3.8 +/- 2.2 | <.0001 |
| Post-Procedure Mitral Regurgitation |  |  |  |  | <.0001 |
| None | 615(1.3%) | 110(1.5%) | 431(1.3%) | 74(1.2%) |  |
| Trace/Trivial | 8007(17.3%) | 1351(18.5%) | 5752(17.6%) | 904(14.3%) |  |
| Mild (1+) | 25880(55.8%) | 4214(57.8%) | 18407(56.2%) | 3259(51.6%) |  |
| Moderate (2+) | 9798(21.1%) | 1381(18.9%) | 6860(20.9%) | 1557(24.6%) |  |
| Mod-Severe (3+) | 740(1.6%) | 95(1.3%) | 502(1.5%) | 143(2.3%) |  |
| Severe (3/4+) | 434(0.9%) | 44(0.6%) | 276(0.8%) | 114(1.8%) |  |
| Severe (4+) | 911(2.0%) | 101(1.4%) | 539(1.6%) | 271(4.3%) |  |
| Cardiac Arrest | 459(0.9%) | 23(0.3%) | 217(0.6%) | 219(3.3%) | <.0001 |
| Cardiac Perforation | 322(0.7%) | 35(0.5%) | 175(0.5%) | 112(1.7%) | <.0001 |
| Cardiac Surgery, unplanned | 664(1.4%) | 47(0.6%) | 345(1.0%) | 272(4.1%) | <.0001 |
| Complete Leaflet Clip Detachment | 45(0.1%) | 5(0.1%) | 30(0.1%) | 10(0.2%) | 0.2110 |
| Single Leaflet Device Attachment | 373(0.8%) | 39(0.5%) | 241(0.7%) | 93(1.4%) | <.0001 |
| Access Site Bleed | 334(0.7%) | 37(0.5%) | 229(0.7%) | 68(1.0%) | 0.0004 |
| In-hospital VARC Degree of Bleeding |  |  |  |  | <.0001 |
| No VARC Bleeding | 47056(97.1%) | 7479(98.2%) | 33416(97.6%) | 6161(93.0%) |  |
| Major Bleed (not life threatening) | 920(1.9%) | 106(1.4%) | 571(1.7%) | 243(3.7%) |  |
| Life threatening or disabling bleed | 488(1.0%) | 28(0.4%) | 238(0.7%) | 222(3.4%) |  |
| Atrial Fibrillation | 595(1.2%) | 50(0.7%) | 340(1.0%) | 205(3.1%) | <.0001 |
| Stroke | 297(0.6%) | 21(0.3%) | 174(0.5%) | 102(1.5%) | <.0001 |
| Post-procedure AKI |  |  |  |  | <.0001 |
| No AKI | 43440(89.6%) | 7613(100.0%) | 32772(95.7%) | 3055(46.1%) |  |
| Stage 1 | 4504(9.3%) | 0(0.0%) | 1296(3.8%) | 3208(48.4%) |  |
| Stage 2 | 86(0.2%) | 0(0.0%) | 0(0.0%) | 86(1.3%) |  |
| Stage 3 | 442(0.9%) | 2(0.0%) | 161(0.5%) | 279(4.2%) |  |
| Dialysis -New Requirement | 315(0.6%) | 2(0.0%) | 108(0.3%) | 205(3.1%) | <.0001 |
| In hospital death | 799(1.6%) | 21(0.3%) | 293(0.9%) | 485(7.3%) | <.0001 |
| Discharge location |  |  |  |  | <.0001 |
| Home | 43524(91.3%) | 7265(95.7%) | 31304(92.2%) | 4955(80.7%) |  |
| Extended Care | 2271(4.8%) | 178(2.3%) | 1474(4.3%) | 619(10.1%) |  |
| Other acute care hospital | 206(0.4%) | 15(0.2%) | 119(0.4%) | 72(1.2%) |  |
| Skilled nursing facility | 1301(2.7%) | 112(1.5%) | 830(2.4%) | 359(5.8%) |  |
| Other | 167(0.4%) | 4(0.1%) | 70(0.2%) | 93(1.5%) |  |
| Left against AMA | 41(0.1%) | 4(0.1%) | 29(0.1%) | 8(0.1%) |  |
| Other discharge location | 162(0.3%) | 16(0.2%) | 110(0.3%) | 36(0.6%) |  |
| Length of stay, days | 4.1 +/- 8.7 | 2.3 +/- 5.6 | 3.7 +/- 8.5 | 8.2 +/- 11.0 | <.0001 |
| * Procedural success is defined as at least 1 device was successfully deployed, procedure was not aborted, relative reduction in MR of at least 1 grade and post procedure mitral regurgitation of none, trace/trivial or mild. | | | | | |

**Supplemental Table 4: Results of Sensitivity Analyses**

|  | Event Rate | Adjusted HR (95% CI) | P value |
| --- | --- | --- | --- |
| **Elective Procedures Only** | | | |
| All-Cause Mortality (1 Year) | | | |
| Improved Renal Function | 11.1% | 0.80 (0.71, 0.90) | < 0.001 |
| Unchanged Renal Function | 15.1% | Reference | -- |
| Worsened Renal Function | 28.7% | 2.85 (2.59, 3.13) | < 0.001 |
| All-Cause Mortality and HF Hospitalization (1 Year) | | | |
| Improved Renal Function | 17.6% | 0.82 (0.75, 0.90) | < 0.001 |
| Unchanged Renal Function | 22.9% | Reference | -- |
| Worsened Renal Function | 36.8% | 2.28 (2.11, 2.47) | < 0.001 |
|  | | | |
| **Model Adjusted for Procedural Complications** | | | |
| All-Cause Mortality (1 Year) | | | |
| Improved Renal Function | 11.9% | 0.78 (0.70, 0.87) | < 0.001 |
| Unchanged Renal Function | 17.2% | Reference | -- |
| Worsened Renal Function | 33.3% | 2.61 (2.40, 2.84) | < 0.001 |
| All-Cause Mortality and HF Hospitalization (1 Year) | | | |
| Improved Renal Function | 18.6% | 0.79 (0.73, 0.87) | < 0.001 |
| Unchanged Renal Function | 25.7% | Reference | -- |
| Worsened Renal Function | 42.3% | 2.14 (1.99, 2.30) | < 0.001 |
